# Particle emissions from heated tobacco products

**DOI:** 10.18332/tpc/185870

**Published:** 2024-04-02

**Authors:** Efthimios N. Zervas, Niki Ε. Matsouki, Chara F. Tsipa, Paraskevi A. Katsaounou

**Affiliations:** 1 Hellenic Open University, Patras, Greece; 2 Medical School, National and Kapodistrian University of Athens, Athens, Greece

**Keywords:** tobacco, public health, novel tobacco products, particle emissions

## Abstract

**INTRODUCTION:**

This study determines the particle emissions from five heated tobacco products (HTPs).

**METHODS:**

An aethalometer is used for the determination of black carbon (BC) and an aerosol monitor for total particulate matter (PM) concentration and also PM fractions (1, 2.5, 4, and 10 μm) in the mainstream emissions of 5 HTPs: IQOS, LIL, PULZE, ILUMA, and GLO. Fifteen different flavors were used, five sticks per flavor, which were smoked using a peristaltic pump under both ISO and Canadian smoking regimes. The method repeatability was determined using 15 sticks of one flavor for each brand for each smoking regime.

**RESULTS:**

All HTPs emit particles, and more than 99.7% of the particles emitted are smaller than 1 μm. Both BC and PM emissions show quite low repeatability. Particle emissions increase in relation to the heating temperature and the intensity smoking regime, and are depending on the flavor used. BC corresponds to a small percentage of total PM.

**CONCLUSIONS:**

Although HTPs are promoted as products of reduced risk compared to conventional cigarettes, high particle concentrations are detected in their emissions, depending on the smoking regime, the flavor used, and the operation parameters. PM emissions vary significantly between different brands under the ISO smoking regime, probably due to the heating temperature. In contrast, PM emissions under the Canadian smoking regime do not vary significantly between different brands. This could probably be attributed to the fact that increased puff frequency does not allow the device to cool down between puffs, resulting in an increase in PM emissions for all the brands, but not dependent on the maximum heating temperature of the device. BC emissions only consist of a very small fraction of PM and do not vary significantly between different brands under both smoking regimes.

## INTRODUCTION

Conventional tobacco products are known for their negative health effects^1^. In the last few years, the tobacco industry and other companies have launched several novel tobacco and related products, such as electronic cigarettes and heated tobacco products (HTPs), which they claim to be ‘less harmful’ than conventional cigarettes (CCs)^2^, despite the limitations of the research^3^. However, it is found that HTPs emit a significant number of toxicants, comparable, in the case of several chemical families, with the number emitted from CCs, though often at lower concentrations^4^. The above findings are mostly based on the data presented by the tobacco industry or from research work funded by it. Moreover, industry-funded research favors the emissions of HTPs over that of independent researchers^5^. It should be noted that HTPs were promoted initially as ‘heat, not burn’ (HNB). However, several studies have clearly shown that incomplete combustion may actually take place^6,7^. This fact leads to the conclusion that combustion pollutants, several of which have severe health effects, are found in the emissions of those products.

Another point to be considered is that several different brands of HTPs are commercialized, with significant differences in heating technology and heating temperature. In addition, several different flavors are available for each kind of device. However, aerosol production depends on various factors, and thus, every type of device should be subjected to detailed studies for the determination of its emissions^8^. Despite these facts, the published data about the emissions so far do not cover all available devices and flavors.

Finally, another parameter of high importance is short commercialization life of those products, which does not allow comprehensive knowledge of their impact, both short-term and long-term, on human health. According to Pisinger et al.^9^, there is insufficient knowledge about the long-term effects of HTPs use; moreover, independently of concentration, these products are found to produce emissions containing toxicants^10^.

The emissions produced from HTPs include a wide variety of chemical species, such as carbonyls, polycyclic aromatic hydrocarbons (PAHs)^11^, metals^12^, aromatic amines^13^, alkanes, organic acids, volatile organic compounds (VOCs)^14^, and particulate matter^6,15^. Particles, depending on their chemical composition in addition to their physical properties, may have severe health effects^16^ and are of primary importance in the study of emissions of tobacco products. The determination of particulate emissions during the use of HTPs has been performed both after their trapping on an appropriate filter and by evaluating the real-time air quality in a room or chamber exposed to HTP emissions.

Total particulate matter (TPM) trapped by a filter under different conditions of use, smoking regimes, ventilation conditions etc., is determined gravimetrically. Concentrations vary from 3.59 to 55.82 mg/item^17,18^. For the indoor air measurements (room or chamber), several techniques are used, such as Condensation Particle Counter (CPC) for the total particle number of sub-micron particles^19^, Fast Mobility Particle Sizer (FMPS)^20^, and Scanning Mobility Particle Sizer (SMPS)^15,21^ for the total number and the particle size distributions in the range of 5.6 to 560 nm^20^. The results for different devices and use conditions present significant variations. Foster et al.^22^ found 6.5–10.3μg/m^3^ PM_1_, 6.6–10.7 μg/m^3^ PM_2.5_, 6.0–12.8 μg/m^3^ PM_10_, during use of THP1.0 under three different ventilation conditions, Mitova et al.^23^ measured both PM_1_ and PM_2.5_ <11 μg/m^3^ during use of THS 2.2 under natural ventilation, and TPM using two different brands, IQOS and GLO, under the same conditions, was 39 (24–127) μg/m^3^ (GLO) and 31 (20–63) μg/m^3^ (IQOS)^24^.

Part of TPM is black carbon (BC), called elemental carbon, soot, light-absorbing carbon, refractory carbon, or graphitic carbon^25^; its concentration is determined using an Aethalometer. Real-time indoor measurements resulted in BC concentrations below the limit of detection at 880 nm and to 0.57 μg/m^3^ at 370 nm^19^, while Savdie et al.^26^ determined 1.18 μg of BC/m^3^ at 880 nm under usual smoking conditions.

However, to the best of our knowledge, and despite the extended commercialization of HTPs, no detailed studies have been performed on real-time emissions of particles during HTPs use. We only found one team of researchers focusing on online analysis of particles emitted from HTPs. According to Wen et al.^21^, particles found were of median diameter around 200–300 nm and of maximum concentration (2–7)×10^8^/cm^3^.

In this study, we examine the following particulate emissions: total PM and several PM fractions (PM_1_, PM_2.5_, PM_4_, and PM_10_), and also BC from several HTPs, using several different flavors under two different smoking regimes (ISO and Canadian). Apart from the concentration of particulate emissions, through the use of several HTPs, we explored the existence of variations among the devices and the influence of the device technology on the emissions. The impact of stick flavor on the concentration of emitted particles was also studied using three different flavors per device.

## METHODS

### Devices and flavors

Five types of commercial HTPs were used in this work: IQOS, ILUMA, LIL (Philip Morris), PULZE (Imperial Tobacco), and GLO (British American Tobacco), with a total of 15 different tobacco flavors. IQOS and ILUMA flavors are Yellow Selection (YS), Silver Selection (SS), and Turquoise Selection (TS); LIL flavors are Regular (R), Roxo (ROX), and Marine (M); PULZE flavors are Capsule Polar (CP), Ice (I) and Rich Bronze (RBr); and GLO flavors are: Classic Tobacco (CT), Arctic Click (AC) and Scarlet Click (SC). It should be noted that IQOS and ILUMA flavored sticks have the same name; however, the sticks of each device are different. The maximum heating temperature of every brand, according to the manufacturers, is 350^o^C for IQOS, ILUMA, and LIL, 345^o^C for PULZE, and 280^o^C for GLO. Moreover, there are differences in heating technology depending on the brand. IQOS, LIL, and PULZE, heat the tobacco by insertion in the center of the stick, of a flat blade, a needle blade, and a cylindrical ceramic rod, respectively, while ILUMA and GLO heat the tobacco stick from the perimeter towards the inside of the stick.

### Emissions generation

**Figure 1:**
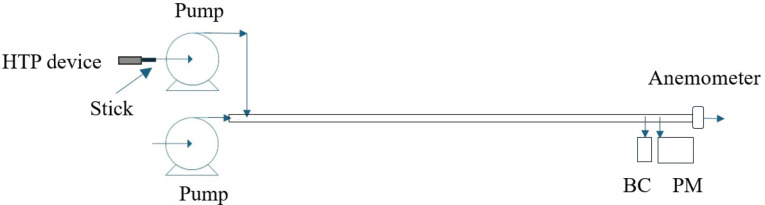
Experimental set-up

Mainstream smoke was generated using a peristaltic pump (Masterflex L/S 07522-20, Cole-Palmer) connected to the HTP device. The device was heated following ISO and Canadian smoking regimes. The pump flow rate was set to 35 mL per puff, and the puff interval was set at 60 s to follow the ISO smoking regime, the pump flow rate was set to 55 mL per puff, and the puff interval was set at 30 s to follow the Canadian smoking regime. The puff duration was 2 s for both regimes. All sticks were smoked until the device was automatically switched off. The number of puffs depends on the device and the smoking regime. Five sticks per flavor were used for each experiment. The repeatability of the method was determined using 15 sticks for one flavor for each device under both ISO and Canadian regimes. The emissions produced with the peristaltic pump were driven through one end of a 3 cm diameter and 1 m length tube to the measurement instruments, which were placed at the other end of the tube. Dilution of the emissions was necessary since undiluted emissions were found to be above the working range of the analytical instruments. Dilution was performed with environmental air using an air pump connected to the same end of the tube with the peristaltic pump. The air velocity produced with the air pump was determined using an anemometer. The dilution factor was 395 ± 8 times for the ISO regime and 251.5 ± 4.5 times for the Canadian one. The experimental setup is presented in Figure 1.

### Particle emissions measurements - instrumentation

Black carbon was measured using a Black Carbon Aethalometer (MicroAethalometer AE51). The resolution of the equipment is 0.001 μgBC/m^3^, and the range is 0–1 mgBC/m^3^. A new filter (T60, Teflon-coated glass fiber filter) was used when the ATN (attenuation) exceeded the value of 100. The instrument was allowed to warm up for 15 min daily. Black Carbon background concentration in indoor air was measured for another 15 min before the samples. Indoor laboratory air was ventilated through the university’s central ventilation system, and no other experiments were performed during the days of the experiments in order to exclude the possibility of particle production from other sources. Moreover, an indoor air cleaner with HEPA filters is constantly functioning in the laboratory to keep particles’ ambient concentration very low. The instrument’s inlet airflow was set to 150 mL/min. Particulate matter concentration in HTP emissions was measured using an Aerosol Monitor (DustTrack DRX 8533). DustTrak Aerosol Monitor is a multi-channel, battery-operated, data-logging device that uses a light-scattering laser photometer that allows the simultaneous measurement of size-segregated mass fraction concentrations corresponding to PM1, PM2.5, PM4, PM10, and Total PM (TPM) size fractions. The resolution of the equipment is ±0.1% of the reading value or 0.001 mg/m^3^, while the range is 0.001–150 mg/m^3^. Prior to every use, a zero-filter calibration was performed according to the manufacturer’s recommendations. Background air was measured for 15 min before each test. The flow rate for this model is fixed to 3.0 L/min. The concentrations of particles in the background air were negligible compared to the particle concentration and were not extracted from the final values. They were used, however, to determine the detection limit of the method. Calculation of the initial concentration of the emitted particles before the dilution was performed based on the values recorded from the analytical instruments. The dilution ratio was determined using an anemometer before every set of experiments.

### Statistical analysis

The outliers per flavor were determined as the values deviating >1 SD (standard deviation) from the mean value, and were excluded for the rest of the analysis. The average percentage of outliers per brand was 24% for GLO, 31% for ILUMA, 36% for LIL, 31% for IQOS, and 37% for PULZE. In order to investigate whether PM and BC concentrations were statistically significant between the different brands, one-way ANOVA statistical analysis was applied for both ISO and Canadian smoking regimes. Results of the ANOVA statistical analysis can be found in the Supplementary file Table 1. Statistical significance was set at p<0.05.

## RESULTS

### Scans

A typical scan of PM fractions, TPM and BC, presents sharp peaks regularly, following every puff (Supplementary file Figure 1). The peaks present a gradual increase, reaching a maximum in the middle of the device’s use time, and then gradually decrease. This trend was observed during all experiments; however, it was less intense in the case of ILUMA. When a peak is not observable, the concentration of particles is below the limit of detection (LOD) of the method. The LOD was calculated as the mean value of the background noise + 3SD. The limit of quantification (LOQ) was calculated as the mean value of the background noise + 9SD.

### Percentage of different PM fractions to total PM

**Figure 2:**
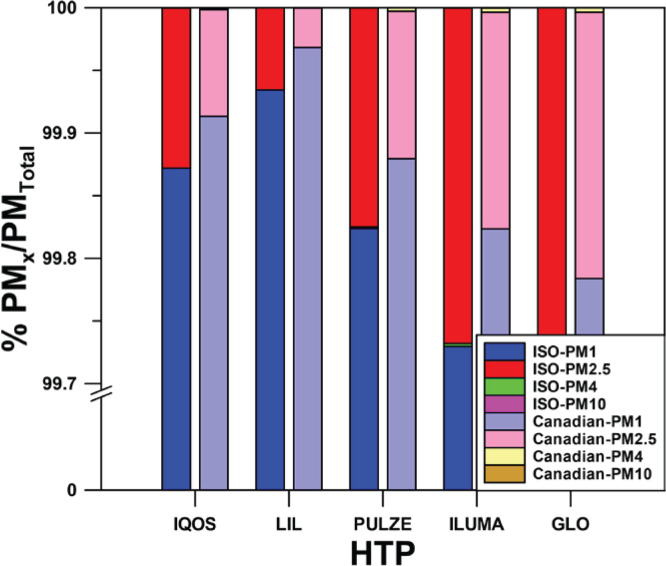
Percentage of different PM fractions over total PM

DustTrak Aerosol monitor can determine several fractions of PM: PM1, PM2.5, PM4, PM10 and Total PM. In all the tests, PM1 corresponded to >99.7% of Total PM. The percentage of PM1 over total PM was 99.87 % in the case of IQOS, 99.93% in the case of LIL, 99.82% for PULZE, 99.72% for ILUMA, and 99.80% for GLO, using the average emissions of all the experiments for the five devices heated under the ISO mode. This percentage is even higher when the devices are used under the Canadian regime (Figure 2). This finding indicates that these devices emit very fine particles <1μm . Only Total PM will be used in the rest of this study, knowing that they correspond to particles of diameter <1μm.

### Mean particle emissions per device

**Figure 3:**
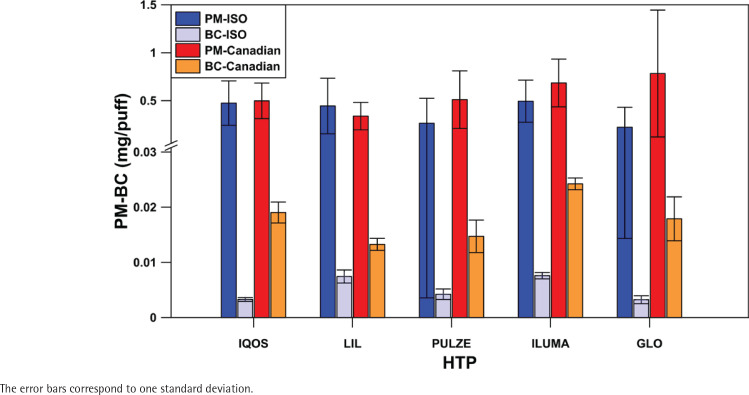
Mean PM and BC emissions per device for ISO and Canadian smoking regimes

Figure 3 shows the total PM and BC mean concentrations of all flavors tested per device, excluding the outliers, both for ISO and Canadian smoking regimes. The corresponding values are 0.474 ± 0.231 mg/puff (ISO) and 0.498 ± 0.186 mg/puff (Canadian) PM for IQOS, 0.444 ± 0.290 mg/puff (ISO) and 0.339 ± 0.144 mg/puff (Canadian) PM for LIL, 0.264 ± 0.260 mg/puff (ISO) and 0.510 ± 0.301 mg/puff (Canadian) PM for PUZLE, 0.494 ± 0.220 mg/puff (ISO) and 0.685 ± 0.249 mg/puff (Canadian) PM for ILUMA, and finally 0.223 ± 0.209 mg/puff (ISO) and 0.783 ± 0.661 mg/puff (Canadian) PM for GLO. BC emissions are 0.00328 ± 0.0035 mg/puff (ISO) and 0.01904 ± 0.00189 mg/puff (Canadian) for IQOS, 0.00744 ± 0.00118 mg/puff (ISO) and 0.01325 ± 0.00109 mg/puff (Canadian) for LIL, 0.00424 ± 0.00096 mg/puff (ISO) and 0.01472 ± 0.00293 mg/puff (Canadian) for PUZLE, 0.00759 ± 0.00058 mg/puff (ISO) and 0.02423 ± 0.00106 mg/puff (Canadian) for ILUMA and 0.00323 ± 0.00073 mg/puff (ISO) and 0.01790 ± 0.00397 mg/ puff (Canadian) in the case of GLO. These results show that BC emissions correspond to a very small fraction of PM: 0.27–0.28% for IQOS, 0.34–0.36% in the case of LIL, 0.29–0.39% for PULZE, 0.27–0.29% for ILUMA, and 0.29–0.31% for GLO.

### Particle emissions per flavor

**Figure 4:**
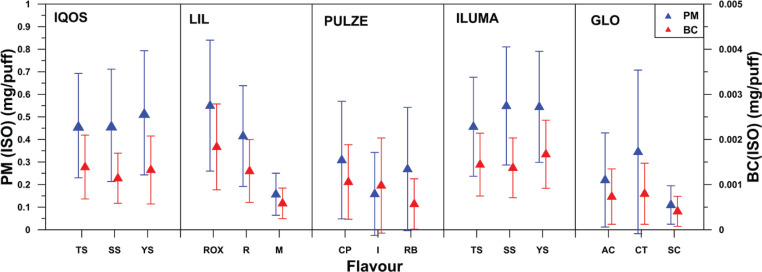
Mean PM and BC emissions per flavor and per device for ISO smoking regimes

**Figure 5:**
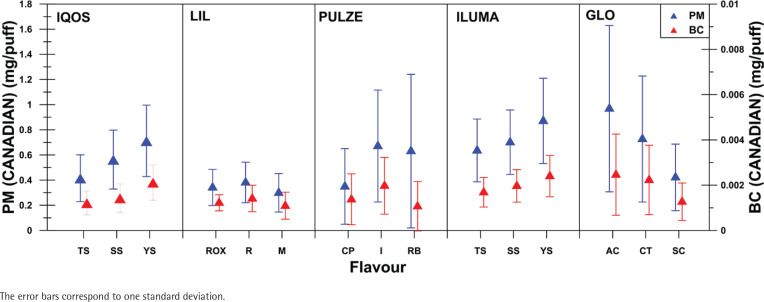
Mean PM and BC emissions per flavor and per device for Canadian smoking regimes

Figures 4 and 5 show PM and BC emissions for all flavors and devices used for ISO and Canadian smoking regimes, respectively. Percent relative standard deviation (%RSD) of the mean PM and BC emissions for the different flavors tested was calculated and was found to have the lowest value for ILUMA, followed by IQOS and then LIL, GLO, and PULZE. The %RSD values for both PM and BC, under ISO and Canadian smoking regimes, varied from 29% to 59% LIL, from 36% to 45% for ILUMA, from 36% to 56% for IQOS, from 62% to 105% for GLO, and from 63% to116% for PULZE.

## DISCUSSION

Particles were detected after heating sticks of 15 different flavors using 5 different devices. Though particles were detected at lower concentrations compared to the emission of conventional cigarettes (about 23.5–44.5% of the concentration of CCs)^27^, the concentration is non-negligible in comparison to that of background air. Raw data of the PM recorded values both for background air and for the five sticks of LIL regular, smoked according to the ISO regime, are presented in Supplementary file Figure 2. As can be seen, under the same conditions, the maximum concentration of PM was 0.03 mg/m^3^ when measurements of background air were performed and 35.5 mg/m^3^, 1183 times higher, during the use of an HTP stick.

PM is already known to be associated with increased mortality and morbidity^28^, and its characteristics, including concentration (measured here), size distribution, and composition (not measured here), seem to determine the extent of the health impact^29^. Particles with a diameter <10 μm can go deep into human lungs or even into the bloodstream, exacerbating respiratory and cardiovascular diseases^30,31^ . Health risks increase as the particles’ size decreases, and particles with diameter <2.5 μm, called fine particles, are considered more dangerous^32^. Our study also showed the presence of particles in HTP emissions. Size-segregated mass size fraction concentration measurements confirmed, as presented in Figure 1, that all the devices emit very fine particles, with a dynamic diameter <1μm, at a percentage higher than 99.7%. This is in accordance with previously published works^24,33^.

In the case of PM emissions per puff, the results show that the ANOVA test is statistically significant (p=0.031) for the ISO smoking regime (Supplementary file Table 1). Therefore, we conclude that there is a significant difference between different brands. To further investigate the statistical significance between the different brands, the results of multiple comparisons (post hoc tests) for each brand are examined. The results for the ISO smoking condition show that ILUMA shows higher PM concentrations than GLO. In contrast, regarding PM in the case of the Canadian smoking regime, the results show that the ANOVA test is not statistically significant (p=0.104). Therefore, there is no difference between different brands.

In the case of BC, the results show that the ANOVA test is also not statistically significant (p>0.05) for both smoking conditions (Supplementary file Table 1). Therefore, we conclude that there is no difference between different brands.

Heating technology was not found to be a parameter determining PM and BC emissions. ILUMA, the new product of IQOS, based on an induction heating system, according to which the sticks are heated from the perimeter to the inside, was not found to emit fewer particles compared to IQOS, LIL, or PULZE, where the sticks are heated from the inside, by using a blade or a pod. In contrast, among devices based on the same heating technology, emissions seem to depend on the maximum heating temperature under the ISO smoking regime. GLO, with a heating temperature of 280^o^C, emits a lower concentration of particles than ILUMA, with a heating temperature of 350^o^C. This could be attributed to the fact that under the ISO regime, all the devices are suspended to repeated cycles of heating and cooling. Applying a puff interval of 60 s allows the devices to cool down after every puff before the temperature increases again, trying to reach the maximum heating temperature of each device (280, 345, and 350^o^C for GLO, PULZE, and IQOS/ILUMA/LIL, respectively). In contrast, when the Canadian smoking regime is followed, the decreased interval between puffs does not allow the devices to cool down after every puff, resulting in no significant difference in PM emissions despite the difference in the brand’s maximum heating temperature. Nevertheless, emissions are dependent on the use parameters (Figure 3), resulting in a higher concentration of particles when the heating conditions are more intense. This increased emissions under the Canadian regime, in comparison to ISO, was not clear in the case of LIL, which may be due to the low repeatability of the method, but, still, the general trend is followed by all the other brands. The quantification of particle emissions of HTPs is characterized by low repeatability, as is the case in PM emissions in combustion systems^34^ and other tobacco products^35^. Additionally, further research is necessary to determine the chemical composition of those emissions.

Concerning the influence of stick flavor on PM emissions, significant variations were found for LIL (ISO) and GLO (Canadian) as presented in Figures 4 and 5. A correlation between flavor and particle concentration and their composition would be a challenge.

Another significant point is that it is often claimed that no combustion occurs in heated tobacco products. The emissions of particles are another argument that combustion indeed occurs^7^.

### Limitations

The emissions shown here are valid under the experimental conditions used and cannot be extrapolated to other conditions. The chemical analysis of particles, which is very important for the evaluation of the toxicity of the particles inhaled, and the measurement of the temperature reached during use, have to be performed, in addition to further evaluation of the toxicity of the emitted particles, so as to compare the emissions of HTPs with the emissions of other tobacco products. Moreover, in future studies, we should take into account the fact that puffing regimes prescribed by ISO and Health Canada do not replicate human behavior.

## CONCLUSIONS

This study focuses on PM and BC emissions during HTP use. Five different devices with 15 different flavors were used. Those sticks produce PM emissions of diameter <1μm in concentrations far above that of the background air. The manufacturers of the devices report different heating temperatures. PM and BC concentration during the use of HTPs increases with the heating temperature of the device. Flavor was found to significantly affect PM and BC emissions. Also, the repeatability of those emissions is quite low and depends on both the device and flavor used. Smoking regime parameters were also found to influence PM and BC emissions. BC emissions corresponded to a quite small percentage of the PMs.

## Supplementary Material



## Data Availability

The data supporting this research are available from the authors on reasonable request.
