# Supplementary material for: Particle emissions from heated tobacco products
Source: Tob Prev Cessat. 2024 Apr 2;10:10.18332/tpc/185870. doi: 10.18332/tpc/185870 (PMC10986040; doi:10.18332/tpc/185870)
Supplement: Supplementary file 1 [file TPC-10-15-s1.pdf]

Supplementary Material

Table 1. Results of the One-way ANOVA test.

ANOVA						
		**Sum of Squares**	**df**	**Mean Square**	**F**	**Sig.**
PM_ISO	Between Groups	0.210	4	0.053	4.142	**0.031**
	Within Groups	0.127	10	0.013		
	Total	0.337	14			
PM_Canadian	Between Groups	0.295	4	0.074	2.560	0.104
	Within Groups	0.288	10	0.029		
	Total	0.584	14			
BC_ISO	Between Groups	0.000	4	0.000	3.163	0.064
	Within Groups	0.000	10	0.000		
	Total	0.000	14			
BC_Canadian	Between Groups	0.000	4	0.000	1.721	0.221
	Within Groups	0.000	10	0.000		
	Total	0.000	14			

Figure 1 presents a typical scan of PM fractions, of Total PM and BC a when a stick (LIL Regular) was heated and puffed under the Canadian regime, following the process described in the Materials and Methods Section. Background air concentration is seen before and after every puff. In the upper diagram, to increase visibility of the results, PM_1, $PM_{2.5}$, PM_4, PM_{10} and PM_{Total}, have been shifted up.

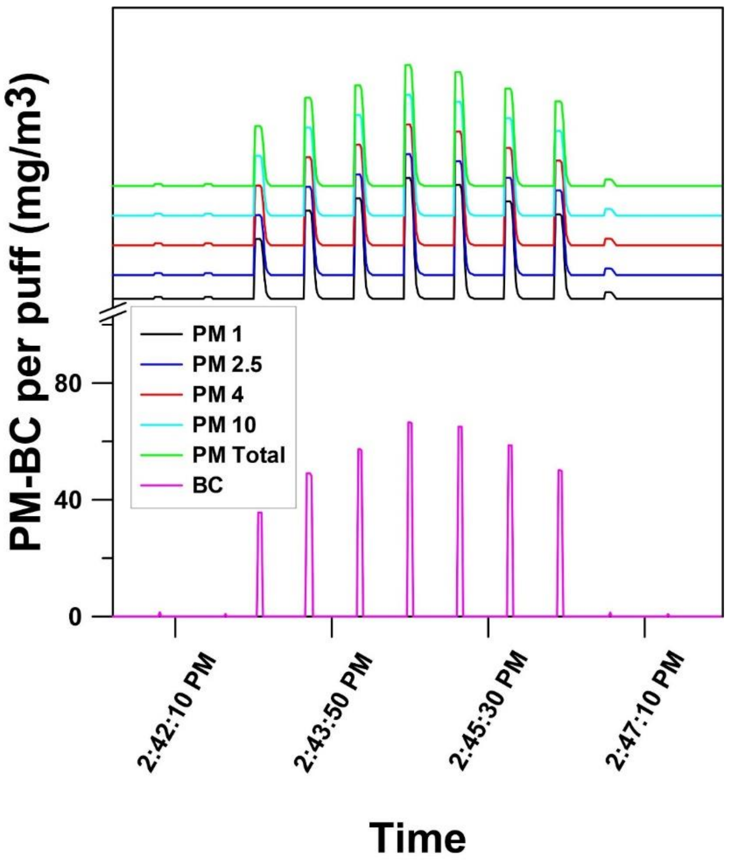

Figure 1. Typical scan of PM fractions and of BC

Figure 2 presents the puff per puff PM results in mg/m^3 for the five sticks of LIL regular under the ISO regime. The increase of PM concentration after every puff can be seen clearly. The scheme on the bottom is the same with the scheme on the top, but we have used different scaling for the y -axis (mg/m^3).

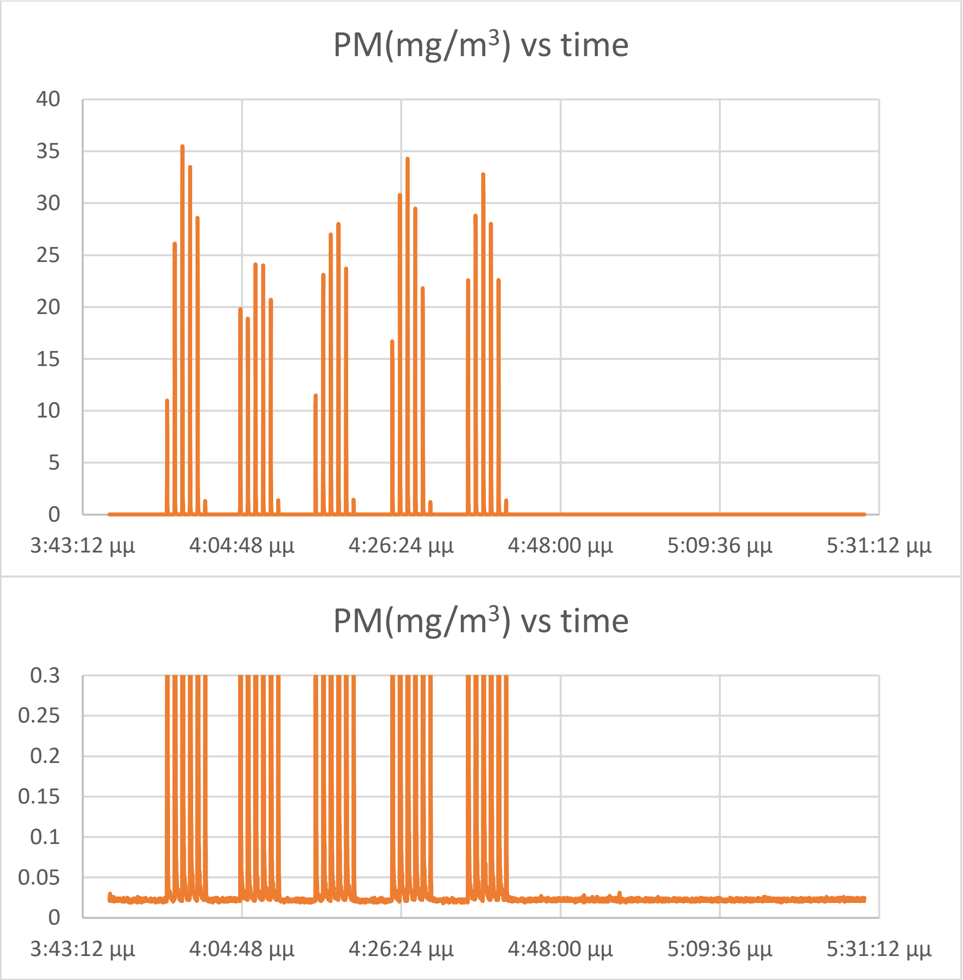

Figure 2. Puff per puff results in mg/m^3 for the five sticks of LIL regular under ISO regime.

© 2024 Zervas E. N. et al.
